# Horses with sustained attention follow the pointing of a human who knows where food is hidden

**DOI:** 10.1038/s41598-021-95727-8

**Published:** 2021-08-10

**Authors:** Monamie Ringhofer, Miléna Trösch, Léa Lansade, Shinya Yamamoto

**Affiliations:** 1 Institute for Advanced Study, Kyoto University, Yoshida Ushinomiya-cho, Sakyo-ku, Kyoto, 6068501 Japan; 2 INRAE, PRC, CNRS, IFCE, Université de Tours, 37380 Nouzilly, France

**Keywords:** Animal behaviour, Zoology

## Abstract

When interacting with humans, domesticated species may respond to communicative gestures, such as pointing. However, it is currently unknown, except for in dogs, if species comprehend the communicative nature of such cues. Here, we investigated whether horses could follow the pointing of a human informant by evaluating the credibility of the information about the food-hiding place provided by the pointing of two informants. Using an object-choice task, we manipulated the attentional state of the two informants during food-hiding events and differentiated their knowledge about the location of the hidden food. Furthermore, we investigated the horses’ visual attention levels towards human behaviour to evaluate the relationship between their motivation and their performance of the task. The result showed that horses that sustained high attention levels could evaluate the credibility of the information and followed the pointing of an informant who knew where food was hidden (Z =  − 2.281, *P* = 0.002, n = 36). This suggests that horses are highly sensitive to the attentional state and pointing gestures of humans, and that they perceive pointing as a communicative cue. This study also indicates that the motivation for the task should be investigated to determine the socio-cognitive abilities of animals.

## Introduction

Obtaining information from other conspecifics is especially important for animals that live in social groups. Domesticated animals who have close interactions with humans in their daily life, should have adapted the ability to obtain interspecies information. To investigate the socio-cognitive abilities of domesticated species, previous studies have tested their interspecific interactions with humans and examined whether they can appropriately respond to human communicative gestures, such as pointing. These studies have shown that several domesticated species can find hidden food by following human pointing in object-choice tasks (dogs^1,2^; horses^3,4^; cats^5^; goats^6,7^; pigs^8^). Among domesticated species, dogs are the most studied and are known to have high sensitivity towards social signals from humans, including pointing. Recent studies have investigated whether dogs choose to evaluate the credibility of the information provided by humans and if they will follow the pointing of a human who has the correct information. These studies revealed that dogs could learn to follow the pointing of an honest human-informant who always pointed to a baited container, and not of a dishonest human-informant who always pointed to an un-baited container^9,10^. Other studies have suggested that dogs can learn to follow the pointing of an informant who has the correct information about the location of hidden-food while referring to the attentional state of two informants during the previous food-baiting event^11,12^. This evidence also indicates that dogs respond to pointing as they comprehend the communicative nature of this cue, which cannot be explained by a simpler mechanism of stimulus enhancement, such as the proximity between the stimulus (herein, pointing) and the target object (herein, hidden food).

Whether domesticated species other than dogs show these abilities, however, is not well understood, and little is known about the evolution of these socio-cognitive mechanisms. To advance this research field, comparative studies with other domesticated animals, such as horses, are essential. Horses have had a close relationship with humans since they were first domesticated approximately 6000 years ago^13–15^. In accordance with this long history of horse–human interactions, recent studies have shown that horses can follow human pointing in object-choice tasks to find the location of hidden-food^3,4^. However, there have been relatively few studies on horses’ socio-cognitive abilities, and to the best of our knowledge, no study has clearly shown that horses can perceive pointing as a communicative cue or evaluate the credibility of the humans’ information provided. To show that horses’ socio-cognitive abilities are equivalent to those of dogs, that is, they comprehend the communicative nature of pointing and are not just applying a simpler mechanism of stimulus enhancement, we need to demonstrate that horses can evaluate human information and choose to follow the pointing of a human according to the human’s knowledge of the correct information (such as the location of hidden food in an object-choice task).

Thus, in this study, we manipulated the credibility of human information by modifying the human informants’ knowledge states and investigated whether horses could follow the pointing after evaluating the credibility of the information. In previous studies, horses were shown to be sensitive to the attentional states of humans^16,17^ and to alter their behaviour according to the previous attentional states of humans^18,19^. Trösch et al.^19^ investigated whether horses were able to alter their begging behaviour, including gazing at and touching, towards two humans by discriminating their attentional states during previous food-hiding events that the horses could observe directly. In the current study, we further investigated the socio-cognitive abilities of horses, examining whether horses discriminate the past attentional states of two humans during the previous food-hiding events that the horses could not directly observe and whether they are able to use the information provided by the pointing of humans and behave accordingly.

We also investigated the relationship between the horses’ visual attention level towards human behaviour during the tests and their performance in the object-choice task. In many previous studies that have tested the cognitive abilities of animals in social contexts, the animals’ poor performance (incorrect choice) has been interpreted as the individuals or species having poor cognitive abilities. Another possibility, however, is that individuals or species simply do not concentrate or pay enough attention to the specific task in the cognitive test^20^. For example, in previous studies on helping behaviour regarding the collaborative tasks of chimpanzees, the limitation in their altruistic helping was thought to be due to their poor cognitive ability to understand the needs of others^21,22^. However, several follow-up studies have suggested that their motivation was more relevant than their socio-cognitive ability^23,24^. The differences in motivation level may cause differences in the performance on the socio-cognitive tests between both individuals and species. The high socio-cognitive abilities of domesticated animals, especially dogs, may also be explained by the high level of attention they pay to humans. For example, dogs have evolved eye contact with humans during the process of domestication^25,26^, which might have resulted in sophisticated socio-cognitive abilities during their interactions with humans. In other words, when the performance of an individual or species on a socio-cognitive test is poor, we should consider several possibilities, especially the following: (1) they cannot do it because of their poor cognitive ability or (2) they can do it but do not because of their low motivation levels.

The aims of our study were (1) to investigate whether horses perceive human pointing as a communicative cue and can follow the pointing of a human who has the information of the food-hiding place by evaluating the credibility of the information and (2) to investigate the relationship between individual attention levels and the performance of horses on the socio-cognitive test using an object-choice task.

## Results

**Figure 1:**
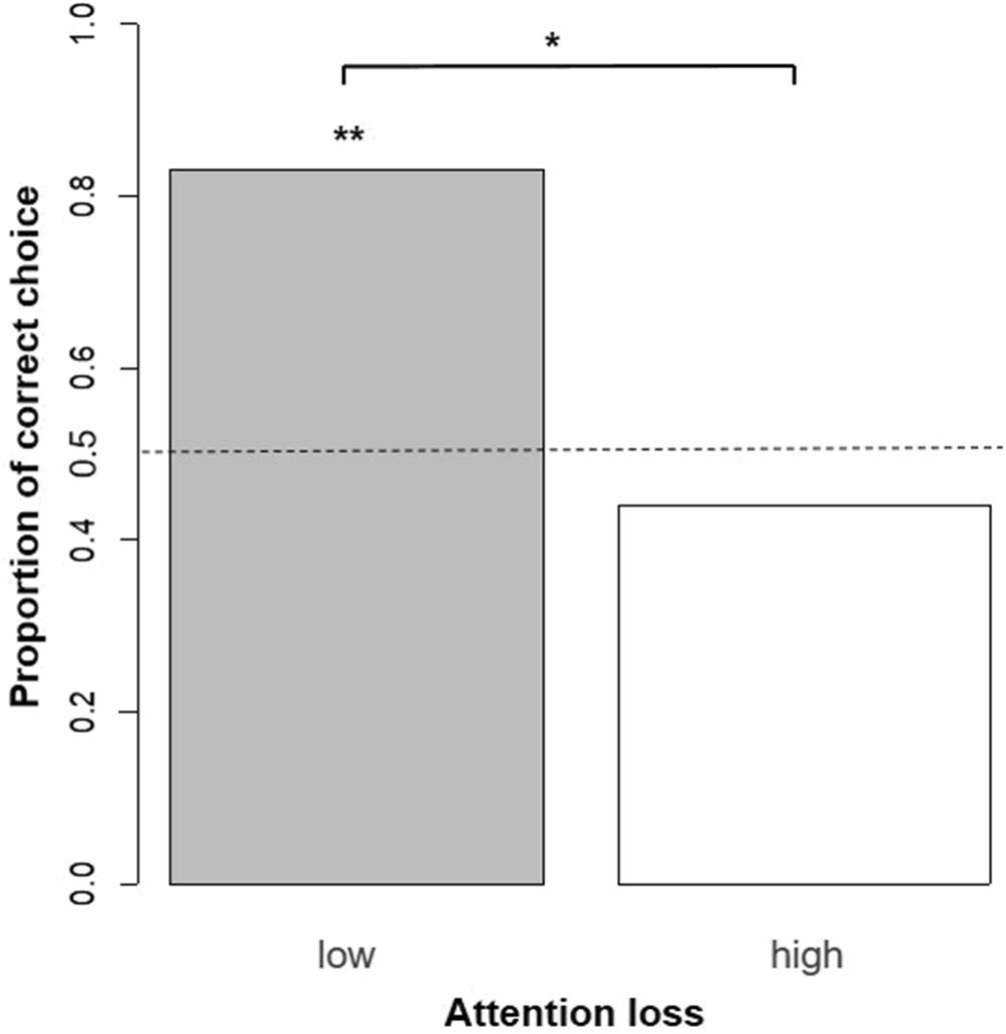
The proportion of correct choices in low and high attention-loss horses. The statistical values indicate the results of the statistical analysis investigating whether the choice was significantly biased compared to a chance level of 0.5 (exact binomial test, n = 18), and investigating whether there were significant differences in the proportion of correct choices of low and high attention-loss horses (Fisher’s exact test, n = 18 for each). **P < 0.01, *P < 0.05.

We tested 54 horses, of which five were excluded as they had problems with habituation (i.e., restless, or afraid of the apparatus) and nine because they did not pass the pre-test. Furthermore, two horses were excluded from the analysis because they stood slightly to the right or left of the middle of the start point, which could bias their choices towards the left or right. Ultimately, we analysed the data from 38 horses who completed the test trial. In the test trial, 25 out of the 38 horses made the correct choice, that is, choosing the knower. Overall, there was a non-significant trend that the horses would make the correct choices following the knower’s pointing (chance level: 0.5; proportion of correct choice = 0.658, exact binomial test, *P* = 0.073, n = 38). Attention loss had a significantly negative effect on the horses’ performance as determined using a generalised linear model (GLM) with binomial error structure and logit link function, conducted with a likelihood ratio test (Z = − 2.281, *P* = 0.002, n = 36); two horses were excluded from this analysis because their videos were not of sufficient quality due to the video camera angle (Table 1). To further analyse the effects of attention loss, we divided the participants into two groups according to the median rate of attention loss (0.08). Horses with low values for attention loss made correct choices significantly more than by chance (0.5; proportion of correct choice = 0.830, exact binomial test, *P* = 0.008, n = 18; Fig. 1), while the correct choices made by those with high values for attention loss were not significantly higher than those by chance (proportion of correct choice = 0.440, exact binomial test, *P* = 0.481, n = 18; Fig. 1). There was a significant difference in the performance between the horses in the high- and low-attention loss groups (Fisher’s exact test, *P* = 0.035; n = 18 for each group; Fig. 1). The side matches did not show any significant effects (Z = − 0.516, *P* = 0.533, n = 36; Table 1).

**Table 1:** Effects of two variables on test performance (correct choice: 1 or incorrect choice: 0) using a generalised linear model with a binomial distribution and logit link function.

Fixed effect	Estimate	Standard error	*X*^*2*^_*1*_	*Z*	*P*
Attention loss	− 14.639	6.417	9.917	− 2.281	0.002
Side matches	− 0.516	0.833	0.388	− 0.619	0.533

Attention loss is the proportion of the duration of attention loss during the total duration of the test trial. Side matches are the value of whether the side of the choice between the last trial in the pre-test and the test trial matched (same sides: 1 or different sides: 0). For the details, refer to the “Materials and methods” section.

## Discussion

This study shows that horses with high sustained attention levels can evaluate the credibility of pointing of human informants, as they made correct decisions in the object-choice task (i.e. they chose to follow the pointing of a reliable informant) based on the attentional state of the informants during a previous food-hiding event. Previous studies in dogs suggested that they can respond to the pointing gestures of humans and comprehend the communicative nature of this cue, and do not just use a simple mechanism of stimulus enhancement, such as close proximity between stimulus (herein, pointing) and the target object (herein, hidden food). Horses have a close relationship with humans similar to that of dogs; however, as of date, there have been much fewer studies in horses compared to those on dogs. Previous studies on horses have shown that they could follow human pointing in an object-choice task to find the location of hidden food^3,4^. The current study adds to the growing body of literature showing that horses could follow the pointing of a reliable human (i.e. the knower, who had the correct information regarding the location of the hidden food) while ignoring the pointing of an unreliable human (i.e. the guesser, who did not have the correct information regarding the location of the hidden food) to find the hidden food in the object-choice task. This suggests that horses may not follow human pointing automatically by the simple mechanism of stimulus enhancement but can recognise it as a communicative cue that contains information from humans like dogs do.

Furthermore, this study provides evidence that horses have high socio-cognitive abilities to understand not only the pointing gesture but also the attentional state of humans. First, the horses could discriminate the attentional state of two humans during the food-hiding event without directly observing where the food was hidden and could follow the pointing of a human who had witnessed the event when they made choices in the object-choice task later on. Previous studies have shown that horses are sensitive to the human attentional state^16,17,27^, and could alter their behaviour according to the past attentional states of humans^18,19^. However, in previous studies on horses using food-hiding events^18,19^, horses could always observe the place where the food was hidden and could recognise the object of interest of the attentive human by using their “eye-object line”^28^ during the food-hiding event. In our study, horses could discriminate the difference in the attentional state of the humans even when there was an obstacle to prevent them from directly observing the location where the food was hidden and the object of interest of humans using the “eye-object line” of humans. Thus, horses seem to be sensitive to the visual perspectives of others (herein, humans), understand that there is a difference between their perspective and others’ perspective^29^, and that an obstacle may prevent their own visual access to the object of interest of others. Second, the horses were presented only one trial per individual, and thus there was no chance for the horse learning the relevancy between the informants’ attentional states and the food; this is different from many previous studies that required many trials per individual to obtain significant results^11,12,30–32^. Thus, horses chose the knower without learning to distinguish between the knower and guesser throughout repeated trials. This indicates that horses had high sensitivity to the humans’ attentional state and could process the situation of the trial immediately. Although further investigations are required to understand the ability of horses to comprehend the mental state of humans, the current study provides further evidence that horses possess some cognitive basis for the ability to understand the knowledge state of humans, and suggests the existence of the basic ability of horses to attribute mental states to humans^33^.

The historical and daily interaction of horses with humans might be the reason for their high socio-cognitive abilities. There are many studies on the socio-cognitive abilities of dogs compared with those for horses, and these studies suggest that the dogs’ sophisticated socio-cognitive abilities towards humans are influenced by the domestication process. Three hypotheses regarding this influence were considered. One is that their lifetime interactions with humans have improved their socio-cognitive abilities towards humans during their ontogenies^34,35^. Second, their high socio-cognitive abilities have evolved through the selection of their socio-cognitive abilities during the domestication process^25,36^. Finally, their high socio-cognitive abilities arose as a by-product of the selection for tameness during the domestication process^37^. Horses have different original ecological traits and domestication processes to dogs, but they also closely interact and cooperatively work with humans. However, there are still few studies on the socio-cognitive abilities of horses compared to those of dogs, and we still do not know which of these three hypotheses can be applied to explain the high socio-cognitive abilities of horses. In order to determine which of the above three hypotheses can be applied to explain the high socio-cognitive abilities of horses, we need more studies investigating the socio-cognitive abilities of horses during their various ontogenetic processes, and comparative studies with relative species living in the wild and forming similar social structures (such as mountain zebra or plains zebra) and other domesticated animals with different original ecological traits and interactions with humans.

The current study also shows that the difference in attention level in individual horses relates to their socio-cognitive abilities towards humans. Individuals with high attention levels towards human behaviour could follow the pointing of a reliable human who has the correct information of the hidden food and could make correct choices in the task (i.e., they showed high socio-cognitive abilities towards humans), whereas those with low attention levels could not. This study showed that the attention level may cause a difference in the performance on the socio-cognitive test between individuals. The difference in the daily interactions and previous experiences with humans might be the reason for the individual differences in attention levels of the horses in the current task. Previous studies in dogs suggested that differences in housing conditions and training could influence an individuals’ gazing behaviour, and thus their attention level towards humans^38–40^. This may also be the case in the horses that participated in this study as they have various backgrounds and may have experienced various kinds of interactions with humans, such as training and housing conditions, as this study was conducted in a riding school. This result implies that the performance on the socio-cognitive test itself does not correctly reflect the socio-cognitive abilities of individuals and species.

In conclusion, we show here that horses are highly sensitive to the attentional state and pointing gestures of humans, and that they perceive pointing as a communicative cue. This study also indicates that the attention level for the cognitive task of individual horses affects their performance of the task. These results, together with those of previous studies^23,24,41^, show the importance of considering the motivation level of individuals/species in socio-cognitive studies in animals.

## Materials and methods

### Ethics statement

Animal husbandry and research protocols for the horses were approved by the Animal Research Ethics Committee of the Wildlife Research Center, Kyoto University (approval number: WRC-2019-004A). This study was carried out in compliance with the ARRIVE guidelines. All procedures adhered to the Japanese Act on the Welfare and Management of Animals.

### Subjects

Fifty-four adult riding horses participated in this study, of which 38 (8 mares and 30 geldings, mean age ± SD, 19 ± 6.14 years; age range 6–27 years; Table S1) completed the test trial and their data were used for the analyses (see “Results” section for details). All horses were kept at the CRANE Riding Club in Osaka, Japan, and ridden daily by members of the riding club. They were housed in individual stalls, fed three times a day, and had ad libitum access to water. No changes were made to their daily care routines in this experiment. All horses were naïve to this type of cognitive experiment.

### Apparatus

**Figure 2:**
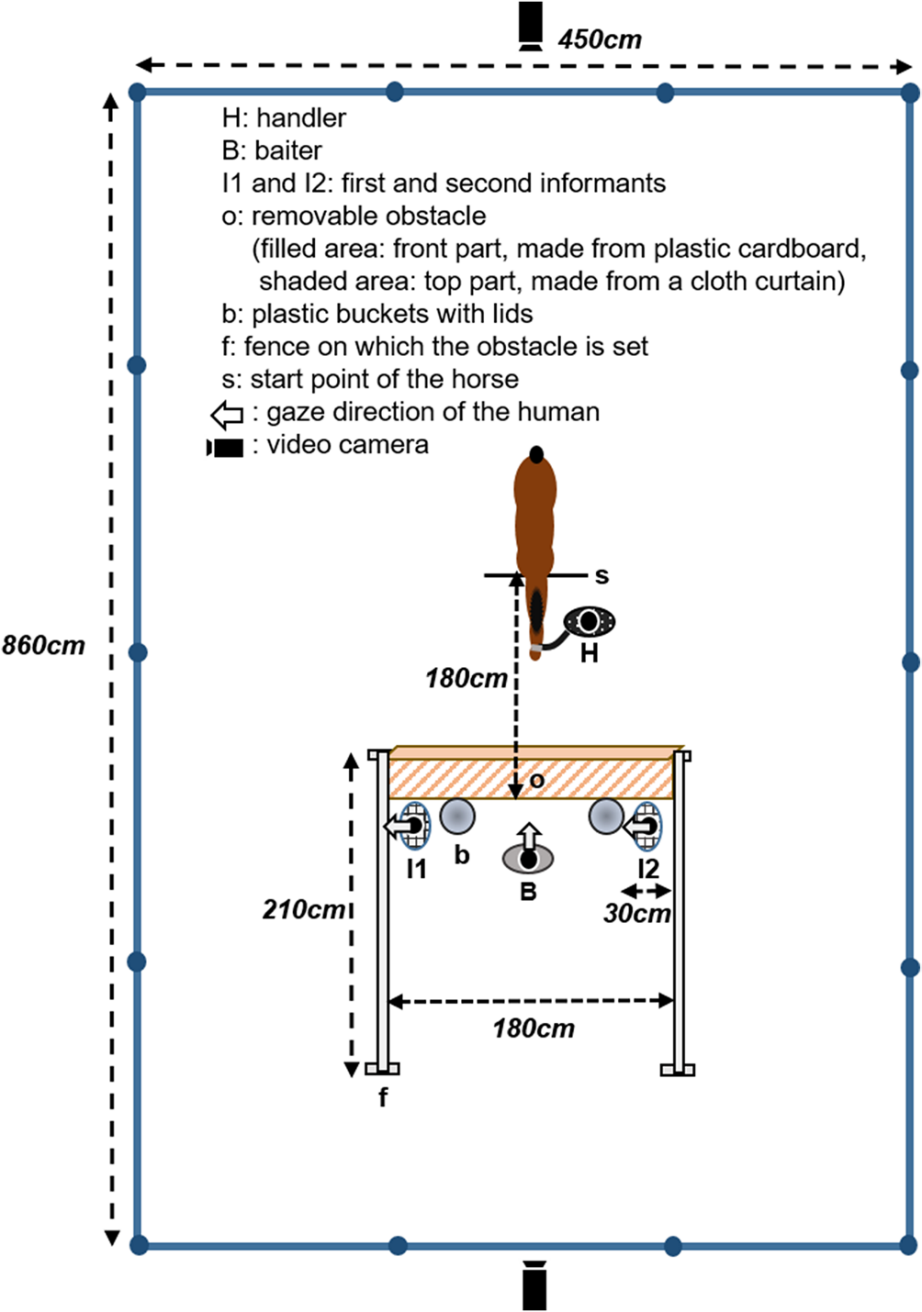
Experimental set-up of the fenced area within a paddock at the beginning of the test trial. Overview of the experimental set-up at the beginning of the test trial. Here, I1 acts as a guesser and I2 acts as a knower.

The horses were tested separately in a fenced area (450 cm × 860 cm) within a familiar paddock (Fig. 2). The experimental apparatus consisted of a removable obstacle on two fences and two plastic buckets with lids behind the obstacle. The food (one piece of carrot) was placed in each bucket prior to the test to prevent the smell of the food in the baited bucket from influencing the horses' choice of bucket. We recorded all experiments using two video cameras (SONY HDR-CX535). Four females conducted the experiments: two as informants (a guesser and a knower), one as a handler of the horses, and one as a baiter (i.e., hiding the food in the bucket). The two informants were unfamiliar with the horse participants, and they acted either as a knower or a guesser depending on the trial.

### Experimental design

We used an object-choice task with two informants, the guesser and the knower, who showed different choices by pointing. The guesser is a human who did not witness the food-hiding event when a baiter hid the food in one of the two closed buckets, that is, the guesser faced away from the baiter and the buckets and did not have the correct information regarding where the food was hidden. The knower is a human who witnessed the food-hiding event by facing toward the baiter and the buckets and had the correct information regarding where the food was hidden. We examined whether horses, ignorant of the food-hiding place, could make a correct choice following the pointing of the human who had the correct information as to where the food was hidden, the knower, by discriminating the past attentional states of the two informants.

### Procedures

Experiments were performed once per horse and consisted of two consecutive steps, the pre-test and the test, before which the horses were familiarised with the set-up for approximately 5 min. The handler walked around inside the fenced area with the horses and allowed them to investigate the apparatus. The baiter then gradually taught the horses to touch the lid of the bucket with their noses to obtain the food. When the horses were calm and had successfully learned to touch the lid of both buckets by themselves, they moved on to the pre-test.

#### Pre-test

The aim of the pre-test was to investigate whether the horses could follow the pointing gesture of a human towards one of the buckets and if they could touch the lid of the bucket to obtain the food (a piece of carrot), without showing any side biases. The pre-test was also used to ensure that the horses understood the experimental procedure. A horse, a handler, and a baiter participated in the pre-test, but the two informants were not present. The pre-test session consisted of the following two steps.

The first step was conducted “without the obstacle” but the other apparatus was identical to that in the test trial. The handler came to a predetermined point (start point in Fig. 2) with the horse and remained stationary. Then, the baiter showed a piece of carrot to the horse and said “Ninjin, ireruyô” in Japanese, meaning “I’ll put the carrot in”. After the baiter opened one of the buckets and placed the carrot inside, the baiter crouched down behind the bucket and pointed to its lid. The baiter pointed with her outside hand from the centre of the apparatus while holding her inside hand behind her back (for example, when the baiter pointed to the lid of the bucket on her right side, she pointed with her right hand while holding her left hand behind her back). Then, when the baiter said “Go!”, the handler released the rope of the horse and allowed the horse to move freely to make a choice. The baiter continued alternating her gaze between the horse and the pointed bucket in one-second intervals, until the horse made a choice. Here, the posture of the baiter was the same as that of the informants during the test trial. All phrases used here were also identical to the phrases used in the following test trial. The trial ended when the horse chose one of the buckets. If the horse did not make a choice within 60 s or walked away from the experimental apparatus, the trial was terminated. The baiters randomly changed the bucket to bait. If the horse made a correct choice (i.e., chose the bucket that the baiter pointed to) in three consecutive trials or four out of five trials, the horse could proceed to the next step. Otherwise, the horse was excluded from the experiment.

The second step was conducted “with the obstacle” to hide the buckets, and the apparatus was identical to that in the test trial. First, the baiter set the obstacle and hid the buckets from the horse’s view. The handler then came to a predetermined place with the horse. The latter procedure was identical to that in the first step (without the obstacle), except for making a sound with the lid of both buckets when closing them and removing the obstacle before the baiter said “Go!”. We intentionally made the closing lid sound to make it difficult for the horses to recognise which bucket was baited and to keep attracting the horse’s interest in this set-up. If the horse made a correct choice (i.e., chose the bucket pointed at by the baiter) in three consecutive trials or four out of five trials, the horse proceeded to the test trial.

#### Test

**Figure 3:**
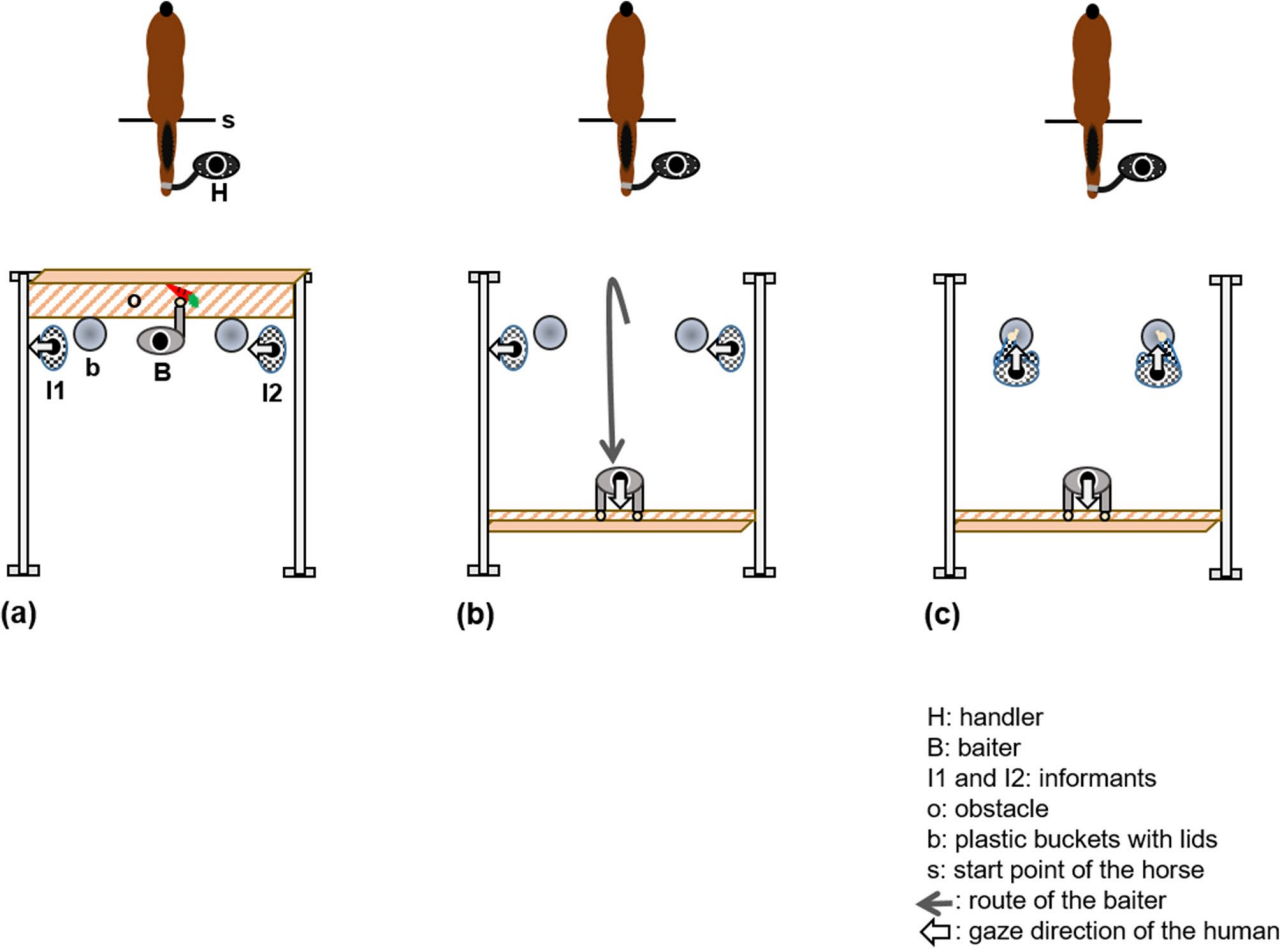
Schematic representation of the test trial. (**a**) When the baiter shows the carrot to the horse. (**b**) When the baiter removes the obstacle and goes into the middle and behind the informants. (**c**) When the two informants go behind the buckets and make choices by pointing at each bucket. Here, I1 acts as a guesser and I2 acts as a knower.

A horse and all four female experimenters (handler, baiter, and two informants) participated in the test trials. The two informants were unfamiliar with the horses prior to the experiment. The informants appeared at the set-up after the cue from the baiter, which was just before the test trial began. After they arrived, they were positioned to predetermined places looking in the same direction with different visual access to the buckets and the baiter (faced/faced away) (Fig. 2). Then, the handler who was blind to the role of the informants came to the start point with the horse and stayed still looking down, so as not to affect the choice of the horse. The baiter showed a whole carrot to the horse and said “Ninjin, ireruyô” in Japanese, meaning “I’ll put the carrot in”. The baiter opened both the buckets placed behind the obstacle and pretended to place the carrot inside the bucket (the carrot was hidden in the front bag of the baiter, the inside of which was invisible), and closed both the buckets, making a sound with the lids (Fig. 3a). While baiting, the horse was unable to observe any of the movements of the baiter that were lower than her chest, because of the obstacle. Then, the baiter stood up, lifted the obstacle, and turned to go to the middle, behind the informants (Fig. 3b). After the baiter said “Go!”, the informants turned using two steps and chose one of the buckets (i.e. crouched down behind one of the buckets and pointed to the lid of each bucket) with the same timing. The informants used their outside hands to point to the bucket lids while holding their inside hands behind their backs (Fig. 3c), to clearly show their choices to the horse. Immediately after the informants started pointing, the baiter said “Go!” for the second time, and the handler released the rope of the horse and allowed the horse to move freely to make a choice. The informants continued alternating their gaze between the horse and the bucket in one-second intervals, until the horse made a choice. When the horse made a choice, it was recorded and the horse’s trial ended. In the test trial, the guesser and the knower participated at the same time and looked in the same direction, behaving identically, and only the visual access of the food-hiding event was different, that is facing towards and away from the buckets and the baiter. We made the behaviour of the informants as uniform as possible because these factors might affect the results of the test trial. To prevent associative learning during continuous trials, we conducted only one trial per horse, as in previous studies^18,19^. The role and sides of the human informants were counterbalanced between the horses to prevent other social factors from having an influence, such as a preference for the human or differences in the pointing confidence of the humans. The sides of the baiting buckets were counterbalanced between the horses.

### Coding and statistical analysis

All experiments were video recorded and coded later. We coded the horses’ choices and the attention of the horses during the test trial. To evaluate the horses’ attention towards the experimental scene (i.e. from the time when the baiter said “Ninjin, ireruyô” until the time when the horse made a choice), we coded their head direction. When the horse was holding its head with its nostrils to the front within 45° angle to the left or to the right^18,42^, we defined the horse as being attentive to the experimental scene in front of it; otherwise, “attention loss” was recorded. We did not consider the horse moving its head to the body or shaking its heads to chase flies away as attention loss. We calculated the proportion of attention loss using the duration of attention loss divided by the total duration of the test trial and then used this value for further analysis. The duration of the test trial was defined as the time between when the baiter first showed the carrot to the horse and said “Ninjin, ireruyô” until the time when the horse touched the lid of one of the buckets and made its choice. A second coder blinded to the aim of this study and conditions of the experiment also coded a random 20% selection of the trials and the inter-observer reliability was sufficient (Spearman’s rank correlation, *r* = 0.83, *P* = 0.018, n = 7) for attention loss. In addition, we coded the horses' choice as a “correct choice” when the horse chose the bucket pointed at by the knower. We also coded whether the side of the choice between the last trial in the pre-test and the test trial matched (hereinafter referred to as side matches). This was because the horses tended to simply choose the side identical to the previous trial when they could not understand the situation and were confused^43,44^.

Statistical analyses were performed using R version 3.5.0^45^. We investigated whether the horses made the correct choice (i.e. choosing the knower) significantly more than the chance level (0.5), using an exact binomial test. To further analyse the results, we focused on two variables: attention loss and side matches (same sides: 1 or different sides: 0). To investigate the effects of the two variables on the results of the test (correct choice: 1 or incorrect choice: 0), we used a GLM with a binomial distribution and logit link function, including the horse’s identity as a random effect. We used a likelihood ratio test to assess the significance of fixed variables.

## Supplementary Information

Supplementary Information.

## Data Availability

Data will be available upon request.
