# Supplementary material for: Horses with sustained attention follow the pointing of a human who knows where food is hidden
Source: Sci Rep. 2021 Aug 10;11:16184. doi: 10.1038/s41598-021-95727-8 (PMC8355342; doi:10.1038/s41598-021-95727-8)
Supplement: Supplementary file 1 — Supplementary Information. [file 41598_2021_95727_MOESM1_ESM.pdf]

Supplementary Information for "Horses with sustained attention follow the pointing of a human who knows where food is hidden"

Authors: Monamie Ringhofer, Miléna Trösch, Léa Lansade, Shinya Yamamoto

Table S1. Information of the 38 horses who completed the test trial.

Name	Sex	Age
Amando	Mare	9
Aren	Gelding	14
Aruno	Gelding	9
Bestogurêdo	Gelding	13
Chiroru	Gelding	23
Curêru	Gelding	22
Diolesansu	Mare	7
Emuesuwan	Gelding	26
Evan	Gelding	22
Fearî	Mare	9
Fureiya	Mare	16
Hebun	Mare	24
Jyueru	Mare	16
Kouryuu	Gelding	19
Kurûzu	Gelding	21
Mahorachûtâ	Gelding	21
Marin	Mare	20
Meipurushirop	Gelding	10
Mistîbarê	Gelding	8
Muramasaô	Gelding	22
Pomushocola	Mare	6
Poraris	Gelding	13
Puraibêtopran	Gelding	15
Puraimu	Gelding	27
Reizâ	Gelding	13
Renjyâ	Gelding	23
Roa	Gelding	14
Sakusesuhyûgô	Gelding	13
Sakusesutacchi	Gelding	16
Sentoerîto	Gelding	24
Shatoru	Gelding	8
Shinborikarento	Gelding	9
Sumairu	Gelding	22
Supiritto	Gelding	23
Tainî	Gelding	26
Taiyoukirakira	Gelding	19
Tanzanaito	Gelding	16
Uranos	Gelding	19
